# Insights into the Prevalence of Software Project Defects

**DOI:** 10.1155/2014/179105

**Published:** 2014-09-07

**Authors:** Javier Alfonso-Cendón, Manuel Castejón Limas, Joaquín B. Ordieres Meré, Juan Pavón

**Affiliations:** ^1^University of Leon, Leon, Spain; ^2^Polytechnic University of Madrid, Madrid, Spain; ^3^Universidad Complutense de Madrid, Madrid, Spain

## Abstract

This paper analyses the effect of the effort distribution along the software development lifecycle on the prevalence of software defects. This analysis is based on data that was collected by the International Software Benchmarking Standards Group (ISBSG) on the development of 4,106 software projects. Data mining techniques have been applied to gain a better understanding of the behaviour of the project activities and to identify a link between the effort distribution and the prevalence of software defects. This analysis has been complemented with the use of a hierarchical clustering algorithm with a dissimilarity based on the likelihood ratio statistic, for exploratory purposes. As a result, different behaviours have been identified for this collection of software development projects, allowing for the definition of risk control strategies to diminish the number and impact of the software defects. It is expected that the use of similar estimations might greatly improve the awareness of project managers on the risks at hand.

## 1. Introduction 

Cost and effort estimation in software projects is a major topic in software engineering research [1]. Nevertheless, in spite of the efforts made, overruns as high as 30% are still commonplace [2]. The frequent failure to develop the project within the targeted cost, schedule, and quality has remarked the need for alternatives to traditional project management. In fact, while most efforts have been focused over the years on traditional project management, very little is yet known about the “actuality” of projects and their management [3].

With a view to improving the quality of their products, project managers are becoming more and more interested in exploiting the benefits gained from a better understanding of their development processes. Amongst the different philosophies to achieve that goal, ISO 9000 standard quality principles recommend adopting a fact-based approach [4]. Irrespectively of the project management methodology the organization responsible for developing the project adheres to, may it be the Prince2 standard, any implementation of the Agile framework, or the PMI best practices covered in the PMBOK Guide [5], the fact-based approach substantially supports the quality of the results obtained in the decision making process. Moreover, IPMA (International Project Management Association) acknowledges in their IPMA Competence Baseline (ICB) [6] the need of management on the different information flows of the project and the potential provided by data warehousing and data mining tools in order to extract hidden knowledge from databases supporting the information systems.

Business managers have for long routinely measured the key variables that describe their businesses while these are in development. Not only storing but also analysing the information recorded in the databases and gaining a deeper understanding of the problems at hand are the next step. Unfortunately, the distillation of useful information might prove problematic as the amount of stored data increases. Eventually, the use of specific tools capable of handling massive data sets becomes mandatory. These tools come from what is known as “data mining,” a discipline that plays a remarkable role at processing and analyzing massive databases such as those found in the business area. Data mining is an active field where researchers make use of tools, techniques, and algorithms from a broad number of disciplines such as artificial intelligence, machine learning, or statistics to name a few.

One of the most interesting applications where data mining tools are making inroads in the software development field is system modeling. The fact that most frequently the relationships amongst process variables are nonlinear and the consequent difficulty to obtain explicit models to describe their behaviour leads to data-based modeling as an improvement oversimplified linear models. Nevertheless, there are still opportunities [7] to improve the results currently obtained.

Data mining tools are useful as well in the description of the behaviour of the processes under study. Exploratory data analysis offers the project manager the opportunity to discover new insights into the processes under development and to confirm or reject common beliefs not supported by the evidence. Amongst the different tools available for exploring the collected data, both supervised and unsupervised classification are active areas of research [8].

Unsupervised classification collects different approaches to establish the models generating the data at hand. The algorithms belonging to this area can be classified as partitive and nonpartitive algorithms. Hierarchical agglomerative cluster analysis in one of the algorithms belongs to the latter family. The results provided by this clustering technique comprehend a complete description of the structure of the processes by providing a global representation of the pairwise dissimilarities amongst the different behaviours observed. This description can be very useful in order to understand how the entire set of data collected can be dissected in smaller structures and the meaning of each of these groups. Moreover, the results can be easily interpreted by using tree graphs.

Supervised classification is a related but significantly different field. The goal pursued is to assign an observation to an already known class. For such purpose, practitioners can make use of a plethora of algorithms, say neural networks, self-organizing maps, or statistical discriminant analysis to name a few. One of the benefits of linear discriminant analysis is that it provides a projection map to visualise the relationships amongst populations generating the data and insights into the relevance of the variables under study for the matter at hand.

This paper makes use of both hierarchical clustering and linear discriminant analysis in order to achieve the following objectives:

- to explore the effect of the effort distribution along the software development lifecycle in the prevalence of the software defects,
- to shed light on those tasks with higher risk levels, as those belonging to the plan and design stages.

This endows project managers and software developers with a powerful tool in order to increase their productivity by reducing software defects along the software development. We describe the material and methods in Section 2 and present the results in Section 3. After a brief discussion in Section 4, Section 5 presents the conclusions.

## 2. Material and Methods

### 2.1. Data under Study

The analysis in this paper is based on the data published by the International Software Benchmarking Standards Group (ISBSG), a nonprofit organization that has created and maintains two repositories of IT historical data (software metrics). The ISBSG has established standards to measure the maintenance, the development, and the enhancement of software. These standards are designed to provide a “common language” of standard terms that can be understood by all professionals [9]. In particular, this paper makes use of a data set that contains information on the development of 4,106 software projects (ISBSG Data CD Release 10). Amongst the different variables contained in the data set, this paper focuses on the total number of defects and on the effort needed during the six phases of the project lifecycle that are considered in the data set: planning, specification, designing, building, testing, and implementation.

A subset of projects was selected from the whole ISBSG data set in order to assure that only those that presented optimal reliability in the data recording method should be used. For such purpose, only those projects with “data quality rating" (this field contains an ISBSG rating code of A, B, C, or D applied to the project data by the ISBSG quality reviewers) and “unadjusted function point rating" (this field contains an ISBSG rating code of A, B, C, or D applied to the functional size (unadjusted function point count) data by the ISBSG quality reviewers) equal to “A” and “count approach" (a description of the technique used to size the project; for most projects in the ISBSG repository this is the functional size measurement method (FSM method) used to measure the functional size (e.g., IFPUG, MARK II, NESMA, FiSMA, COSMIC-FFP, etc.)) equal to “IFPUG” were considered [9]. Those projects with no data recorded on the effort variables were discarded as well. The subset after applying this filter contained information about 301 projects.

### 2.2. Hierarchical Clustering Analysis

In order to represent the whole structure of pairwise dissimilarities amongst the effort distribution of the different projects, we applied the hierarchical clustering algorithm proposed by Ciampi et al. [10]. This work presents a general algorithm of hierarchical agglomerative clustering that spawns into a variety of concrete specifications. In particular, they develop the models related to some familiar distributions of the exponential family (multivariate normal, Poisson, and multinomial) and a particularly useful two-step algorithm for handling the analysis of massive data sets.

In essence, this general clustering algorithm specifies a dissimilarity based on the likelihood ratio statistics and an agglomeration rule that, by successive iterations, merges the different observations according to their dissimilarity, substituting each pair of merged subpopulations by their union until all subpopulations have been merged.

For that purpose, it is supposed that the data set contains a number *M* of subpopulations *P*_1_, *P*_2_,…, *P*_*M*_, that each data set is an independent random sample from the corresponding subpopulation, and that the distribution of each population is known except for an unknown parameter. Thus each subpopulation is represented by its distribution: *f*_1_(*x*∣*θ*_1_), *f*_2_(*x*∣*θ*_2_),…, *f*_*M*_(*x*∣*θ*_*M*_).

In such context, the dissimilarity amongst two different subpopulations can be defined in terms of the likelihood ratio statistic to test the hypothesis *H*_0_ that two subpopulations have the same parameter against the hypothesis *H*_1_ that the two parameters are different. This definition expresses the idea that two subpopulations are similar to the extent that they cannot be easily distinguished statistically:

(1) $$\begin{array}{c} d\left(P_{i}+P_{j}\right)=2\ln\frac{{\hat{L}}_{1}}{{\hat{L}}_{2}}. \end{array}$$

Following a similar approach, Ciampi et al. [10] recalculate the dissimilarity between the merged pair and all other subpopulations by means of a model-based agglomeration rule that defines the dissimilarity between two aggregates *A*_*i*_ and *A*_*j*_ as

(2) $$\begin{array}{c} d\left(A_{i}+A_{j}\right)=2\ln\frac{{\hat{L}}_{1}}{{\hat{L}}_{2}}. \end{array}$$

The detailed formulas for calculating the dissimilarity between atoms and the dissimilarity between aggregates must be developed according to the distributions of the subpopulations. For the purpose of analysing the distribution of the effort along the project lifecycle the data set under study can be viewed as a contingency table whose observations follow the multinomial distribution *M*(*p*_*r*_, *N*_*r*_), where *N*_*r*_ is known for each *p*_*r*_. In this particular case, for the multinomial distribution the general algorithm is specified as

(3) $$\begin{array}{c} d_{M}\left(A_{i}+A_{j}\right)=\overset{p}{\underset{l=1}{\sum }}\left[n_{il}{\ln\hat{p}}_{\mathrm{il}}+n_{jl}{\ln\hat{p}}_{jl}-\left(n_{il}+n_{jl}\right)\ln{\hat{p}}_{i\cup jl}\right], \end{array}$$

where *n*_*rl*_ represent the *l*th component of the *r*th sample (*r* = *i*, *j*) and ${\hat{p}}_{i\cup jl}=(n_{il}+n_{jl})/(N_{i}+N_{j}),\;\;{\hat{p}}_{gl}=n_{gl}/N_{g}$ are the components of the maximum likelihood estimators of the *p*_*r*_ parameters (*r* = *i*, *j*, *i* ∪ *j*).

### 2.3. Discriminant Analysis

The main purpose of the discriminant analysis techniques is to provide a set of rules to predict the classes to which the different observations belong. Nevertheless, specific techniques like the linear discriminant analysis [11] may serve as a complementary tool to support the understanding of the results of the cluster analysis.

Linear discriminant analysis can be seen as well as a linear projector which provides maps to visualize the layout of the observations on a reduced dimension. This projection is calculated according to the criteria of obtaining that projection where the different classes are rendered as far as possible from each other and where the data of each class is as concentrated as possible. This kind of projection is very suitable to visualize the results of a clustering algorithm to confirm whether or not the different clusters overlap.

Additionally, linear discriminant analysis, as a linear projector, is based on a change of basis matrix which may provide relevant information on which variables have the largest influence on the separation of the classes.

This analysis uses LDA for both purposes in the exploration of the structure of the data set.

## 3. Results of the Analysis

**Figure 1:**
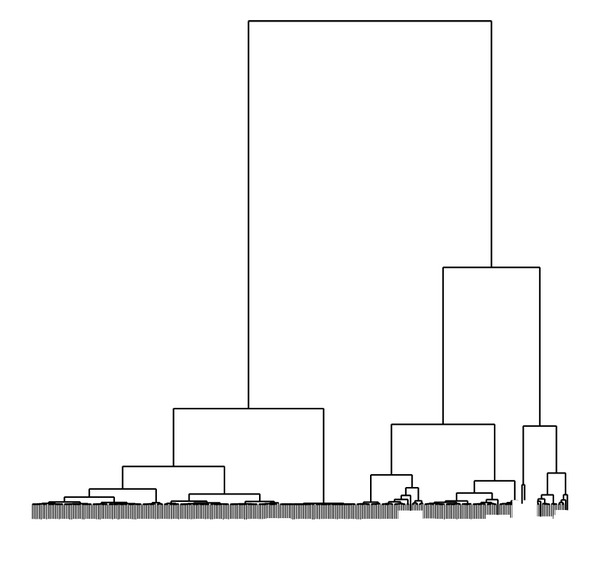
Dendrogram obtained by applying the multinomial specification of the clustering algorithm.

**Figure 2:**
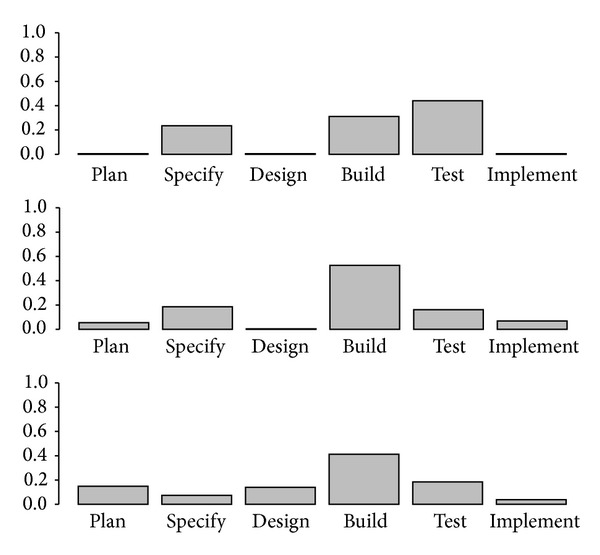
Profile of the mean vectors for the three classes identified.

Figure 1 shows the hierarchical tree of relationships obtained by applying the clustering algorithm proposed by Ciampi et al. [10] using the multinomial specification on the effort related variables. How the different observations at the bottom are agglomerated pairwise into larger structures that are separated to an extent that is proportional to the height at which their agglomeration is represented can be seen. Thus, Figure 1 shows a large number of observations at the bottom that successively agglomerate at increasing heights as larger groups are obtained. At the highest steps, just a few large groups remain to be joined and their separation can be appreciated as significant. This tree suggests that at least three main different behaviours can be identified. These classes can be characterised by means of their mean vectors which are represented in Figure 2. These profiles show significant differences on the mean value of the effort variables for each class, which suggests that relevant differences amongst them might be found.

**Figure 3:**
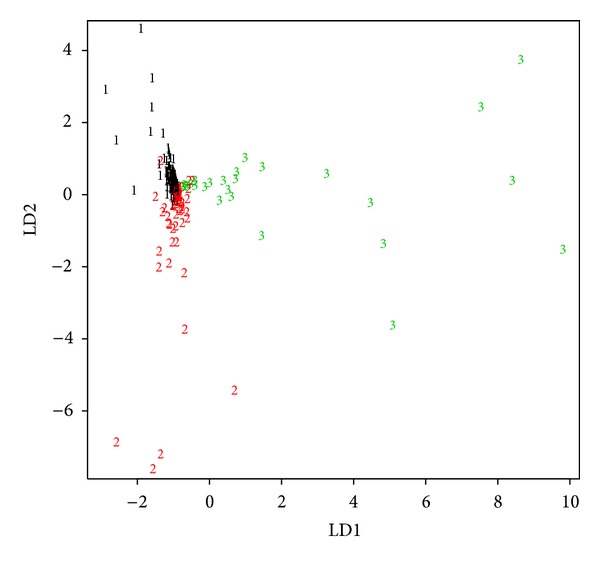
Linear discriminant analysis projection on the first (LD1) and second (LD2) discriminant functions.

The linear discriminant analysis provided both a projection map (Figure 3) and a change of basis matrix (shown in Table 1 with percentage values). The projection map confirms the existence of the three classes while it shows that they are partially overlapped. The change of basis matrix shows that the efforts during the design (73.44%) and plan (9.53%) are the variables with greater influence on the separation of the classes according to the first discriminant function. It also shows that the efforts during the implementation and test are the variables with greater influence on the separation of the classes according to the second discriminant function.

The results formerly shown only describe the structure of the data set in terms of the effort variables and the different classes that have been identified and profiled. Nevertheless, no relationship has been drawn with the defects prevalence as the “total defects delivered” variable has not been considered yet. In order to examine the relationship between the different effort profiles and the defects prevalence we consider the ratio of faulty projects (those with values of the “total defects delivered” greater than zero) within each class (Table 2).

The values shown in Table 2 display significant differences of the ratio of faulty projects on each class.

The results obtained show significant differences on the defects prevalence amongst the classes identified by means of the hierarchical clustering algorithm. The effort needed during the design and implementation was, for the projects under study, the factor with greater impact on the defects prevalence. Those projects with higher needs during these two phases rendered more defects than those with greater loads along the other phases.

These results agree with the studies performed by TRW, Nippon Electric, and Mitre Corp. [12] that state that design activities introduce from 50% to 60% of the total defects along software development. This is an aspect reinforced by further studies [13] that consider the defects during this phase as those that programmers must address in order to increase their productivity and reliability so as to reduce the defects amplification issues described by [14]. Even though classic textbooks like Pressman and the studies formerly mentioned already considered these aspects amongst the most influential ones, this paper provides another perspective to this antique problem difficult to tackle with a novel approach based on actual data from developed projects [9] and the use of modern techniques of hierarchical clustering [10] and data analysis.

## 4. Discussion

It can be argued that the dendrogram provided by the hierarchical clustering algorithm suggests the existence of two main different classes. There is no contradiction with the results obtained by choosing three main different classes as classes 2 and 3 show a somewhat similar profile and the results shown in Table 2 are quite similar giving both of them the higher prevalence ratios. We preferred to choose three classes instead of just only two because the projection map obtained by the linear discriminant analysis with three classes provided a clearer picture and a better separation of the identified classes.

## 5. Conclusions

The use of a fact-based approach has proven useful in extracting hidden information related to the development of software projects. Data mining techniques such as supervised and unsupervised classification successfully described the internal structure of the data in order to provide useful results. The projects under study were classified into three classes whose profiles have shown relevant differences on the mean value of the effort at each stage of the development process. The efforts during the design (73.44%) and plan (9.53%) are the variables with greater influence on the classification. The effort needed during the design and implementation was, for the projects under study, the factors with greater impact on the defects prevalence. Those projects with higher needs during these two phases rendered more defects than those with greater loads along the other phases.

Project managers are encouraged to use a similar approach on their own data in order to extract their own conclusions in the context of their business environment.

## Figures and Tables

**Table 1:** Contribution of the effort related variables on the first (LD1) and second (LD2) discriminant functions.

	LD1	LD2
Plan	9.53%	5.93%
Specify	3.27%	2.15%
Design	73.44%	18.06%
Build	2.19%	7.48%
Test	2.94%	19.55%
Implement	8.63%	46.83%

**Table 2:** Defects prevalence on each of the three identified classes.

	Class 1	Class 2	Class 3
Faulty projects	15.38%	21.52%	37.03%
